# Supplementary material for: 16S ribosomal RNA modification drives transcript-specific translation efficiency
Source: bioRxiv. 2026 Apr 20:2026.04.20.719615. Preprint. [Version 1] doi: 10.64898/2026.04.20.719615 (PMC13317604; doi:10.64898/2026.04.20.719615)
Supplement: Supplement 1 [file NIHPP2026.04.20.719615v1-supplement-1.pdf]

bioRxiv preprint doi: https://doi.org/10.64898/2026.04.20.719615; this version posted April 20, 2026. The copyright holder for this preprint (which was not certified by peer review) is the author/funder. This article is a US Government work. It is not subject to copyright under 17 USC 105 and is also made available for use under a CC0 license.

SUPPLEMENTAL INFORMATION

16S ribosomal RNA modification drives transcript-specific translation efficiency

Zachory M. Park, Christina R. Savage, Amanda R. Decker, Chin-Hsien Tai, Tapan K. Maity, Weiming Yang, Lisa M. Jenkins, and Kumaran S. Ramamurthi

Contents:

Supplemental Figures S1-S7

Supplemental Table S1

bioRxiv preprint doi: https://doi.org/10.64898/2026.04.20.719615; this version posted April 20, 2026. The copyright holder for this preprint (which was not certified by peer review) is the author/funder. This article is a US Government work. It is not subject to copyright under 17 USC 105 and is also made available for use under a CC0 license.

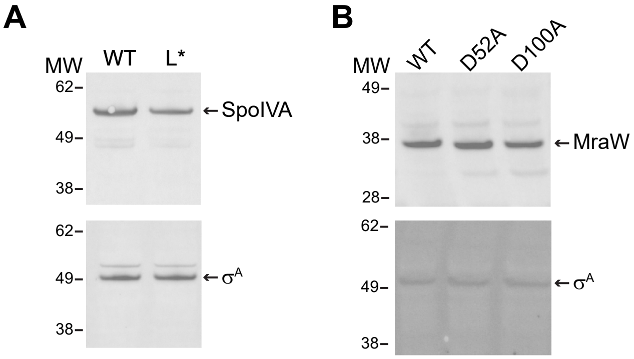

Figure S1. SpoIVA^{L59P} and MraW* are expressed at levels comparable to wild type versions of the proteins. (A) Cell extracts of sporulating *B. subtilis* producing either WT SpoIVA or SpoIVA^{L*} harvested 2.5 h after induction of sporulation examined by immunoblotting using antisera to detect (top) SpoIVA or (bottom) SigA used as a loading control. Strains: KR394 and ZP47. (B) Cell extracts of sporulating *B. subtilis* producing either WT MraW, MraW^{D52A}, or MraW^{D100A} harvested 4 h after induction of sporulation examined by immunoblotting using antisera to detect (top) MraW or (bottom) SigA. Strains: ZP134, ZP231, and ZP233.

bioRxiv preprint doi: https://doi.org/10.64898/2026.04.20.719615; this version posted April 20, 2026. The copyright holder for this preprint (which was not certified by peer review) is the author/funder. This article is a US Government work. It is not subject to copyright under 17 USC 105 and is also made available for use under a CC0 license.

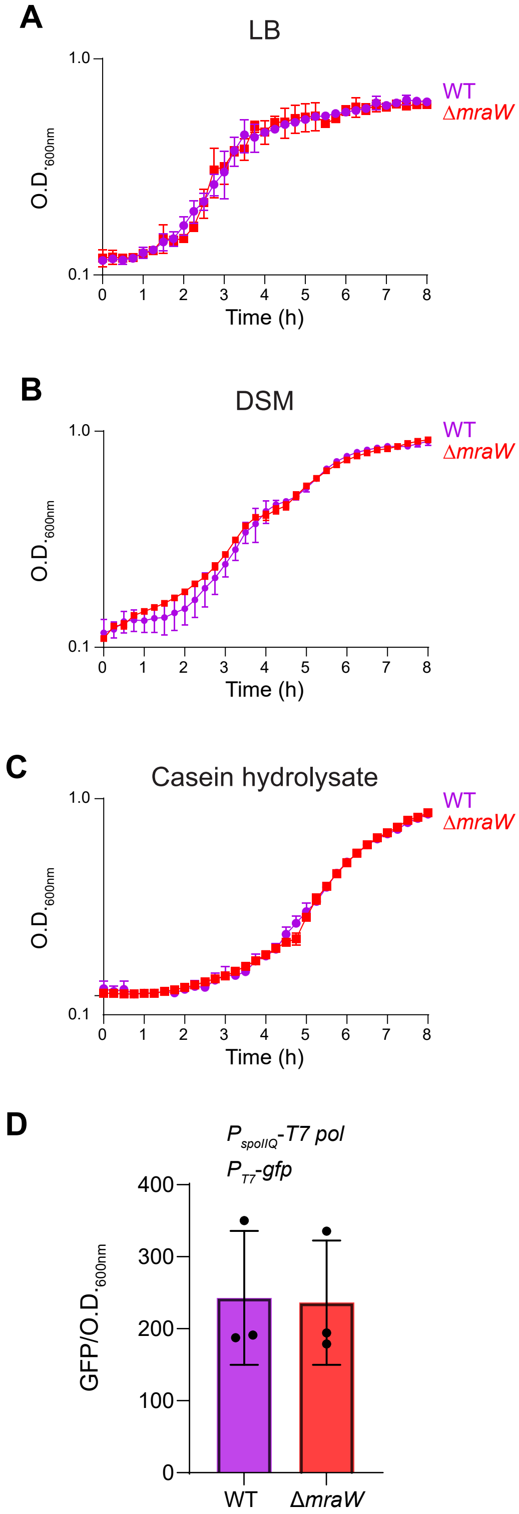

Figure S2. Growth rate of *B. subtilis* in several growth media is unaffected by deletion of *mraW*. Growth assayed by optical density measured at 600 nm (O.D.$_{600nm}$) at indicated time points of WT (purple) or Δ*mraW* cells grown in (A) lysogeny broth (LB), (B) Difco sporulation medium (DSM), or (C) casein hydrolysate medium. Data points represent mean (n = 3 independent cultures); errors: S.D. (D) Production of GFP in vivo whose gene expression was driven from a T7 promoter with a 5' UTR that was identical to the template used in the in vitro transcription and translation assay in Figure 1A. Samples were analyzed after 18 hours of growth in LB 10/10/10 to allow for activation of the σ^F-dependent *spoIIQ* promoter that was driving T7 polymerase expression. Data points represent mean (n = 3 independent cultures); errors: S.D. Strains ZP572 and ZP574.

bioRxiv preprint doi: https://doi.org/10.64898/2026.04.20.719615; this version posted April 20, 2026. The copyright holder for this preprint (which was not certified by peer review) is the author/funder. This article is a US Government work. It is not subject to copyright under 17 USC 105 and is also made available for use under a CC0 license.

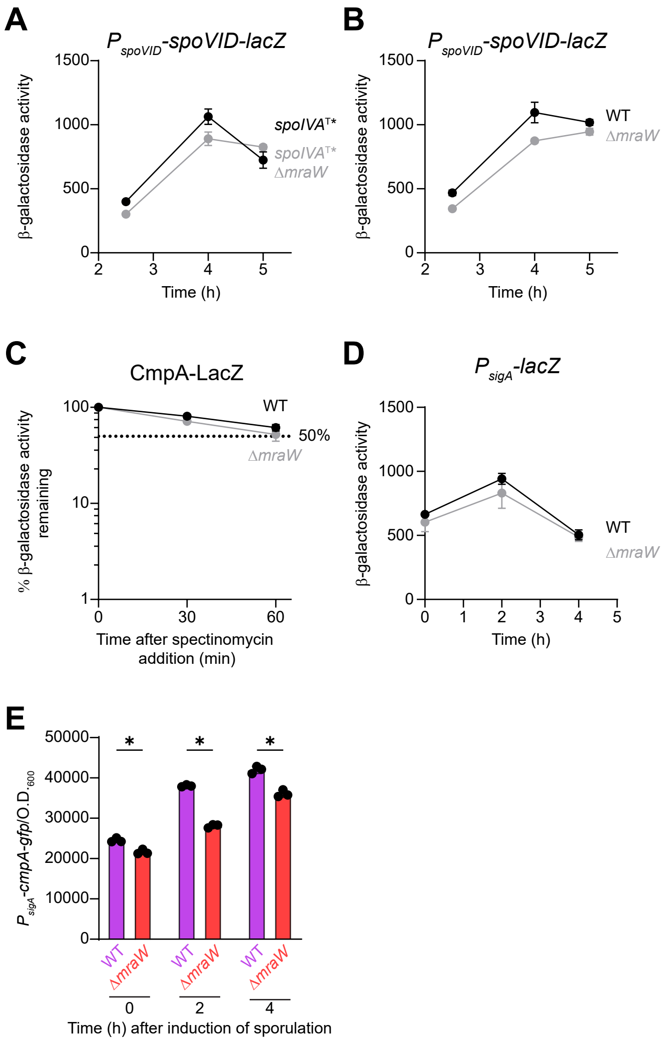

Figure S3. SpoVID production, CmpA turn-over rate, and *lacZ* expression, and are not impacted in Δ*mraW*. (A-B) β-galactosidase activity arising from translational fusion of the *spoVID* ORF to *lacZ* expressed from the *spoVID* promoter at indicated time points after induction of sporulation in the (black) presence or (gray) absence of *mraW* in cells harboring (A) *spoIVA*^{T*} or (B) WT *spoIVA*. Strains ZP405, ZP410, ZP339, ZP345. (C) β-galactosidase activity arising from translational fusion of *cmpA* ORF to *lacZ* after addition of 200μg mL-1 spectinomycin added 5.5 h after induction of sporulation to arrest translation in the (black) presence or (gray) absence of *mraW*. Activity is reported as % β-galactosidase activity remaining relative to t = 0. Data points represent mean (n = 3 independent cultures); errors: S.D. Strains SE230 and ZP313. (D) β-galactosidase activity arising from a transcriptional fusion of a constitutive promoter regulated by σ^A to *lacZ* ORF at indicated time points after induction of sporulation in the (black) presence or (gray) absence of *mraW*. Strains ZP363 and ZP361. Bars represent mean (n = 3 biological replicates); errors: S.D. (E) Levels of fluorescence generated by a CmpA-GFP fusion produced from a constitutive promoter regulated by σ^A relative to culture density ($O.D._{600nm}$) at indicated time points after induction of sporulation in the (purple) presence or (red) absence of mraW. Statistical analysis: unpaired t-tests with the Bonferroni-Dunn correction for multiple comparisons, * indicates P-value < 0.05. Bars represent mean (n = 3 biological replicates); errors: S.D. Strains: ZP523 and ZP525.

bioRxiv preprint doi: https://doi.org/10.64898/2026.04.20.719615; this version posted April 20, 2026. The copyright holder for this preprint (which was not certified by peer review) is the author/funder. This article is a US Government work. It is not subject to copyright under 17 USC 105 and is also made available for use under a CC0 license.

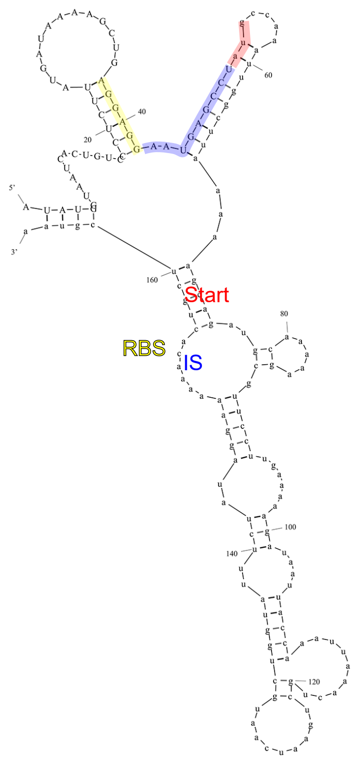

Figure S4. A stem-loop structure is predicted to occlude the start codon of *cmpA* mRNAs. Mfold prediction of the full length *cmpA* mRNA. The RBS is highlighted in yellow, the intervening sequence (IS) between the RBS and start is highlighted in blue, and the start codon (AUG) is highlighted in red. Sequence in uppercase represents the 5'UTR of *cmpA* mRNA and lowercase represents the *cmpA* coding sequence.

bioRxiv preprint doi: https://doi.org/10.64898/2026.04.20.719615; this version posted April 20, 2026. The copyright holder for this preprint (which was not certified by peer review) is the author/funder. This article is a US Government work. It is not subject to copyright under 17 USC 105 and is also made available for use under a CC0 license.

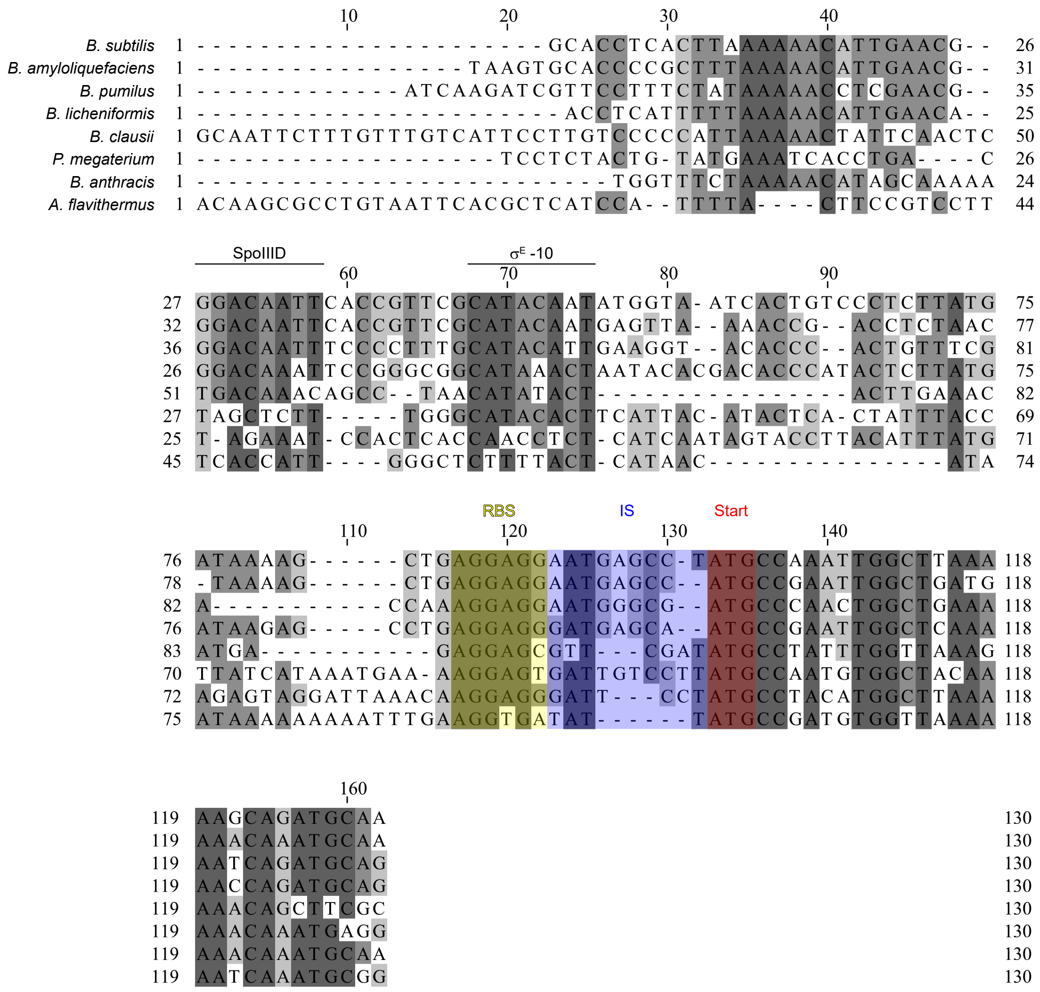

Figure S5. Sequence conservation of the *cmpA* RBS, IS, and first several codons of the open reading frame across related species. Sequence conservation of the upstream region and first 10 codons of *cmpA* across various species: *Bacillus subtilis* (NC_000964.3), *Bacillus amyloliquefaciens* (NZ_CP072120.1), *Bacillus pumilus* (NC_009848.4), *Bacillus licheniformis* (NZ_CP140161.1), *Bacillus clausii* (NZ_CP140150.1), *Priestia megaterium* (NZ_CP035094.1), *Bacillus anthracis* (NC_007530.2), and *Anoxybacillus flavithermus* (NC_011567.1). The RBS is highlighted in yellow, the IS is highlighted in blue, and the start codon is highlighted in red.

bioRxiv preprint doi: https://doi.org/10.64898/2026.04.20.719615; this version posted April 20, 2026. The copyright holder for this preprint (which was not certified by peer review) is the author/funder. This article is a US Government work. It is not subject to copyright under 17 USC 105 and is also made available for use under a CC0 license.

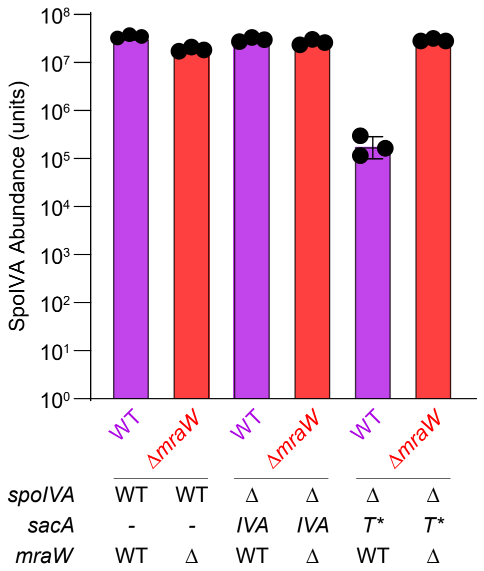

Figure S6. SpoIVA^{T}* levels are restored to wild type levels in Δ*mraW*. SpoIVA abundance measured by mass-spectrometry in strains harboring the indicated allele of *spoIVA* in the presence and absence of *mraW*. *sacA* is a chromosomal locus from which the indicated allele of *spoIVA* is expressed. Strains: PY79, ZP1, ZP429, ZP435, ZP379, and ZP381. Bars represent mean (n = 3 biological replicates); errors: S.D.

bioRxiv preprint doi: https://doi.org/10.64898/2026.04.20.719615; this version posted April 20, 2026. The copyright holder for this preprint (which was not certified by peer review) is the author/funder. This article is a US Government work. It is not subject to copyright under 17 USC 105 and is also made available for use under a CC0 license.

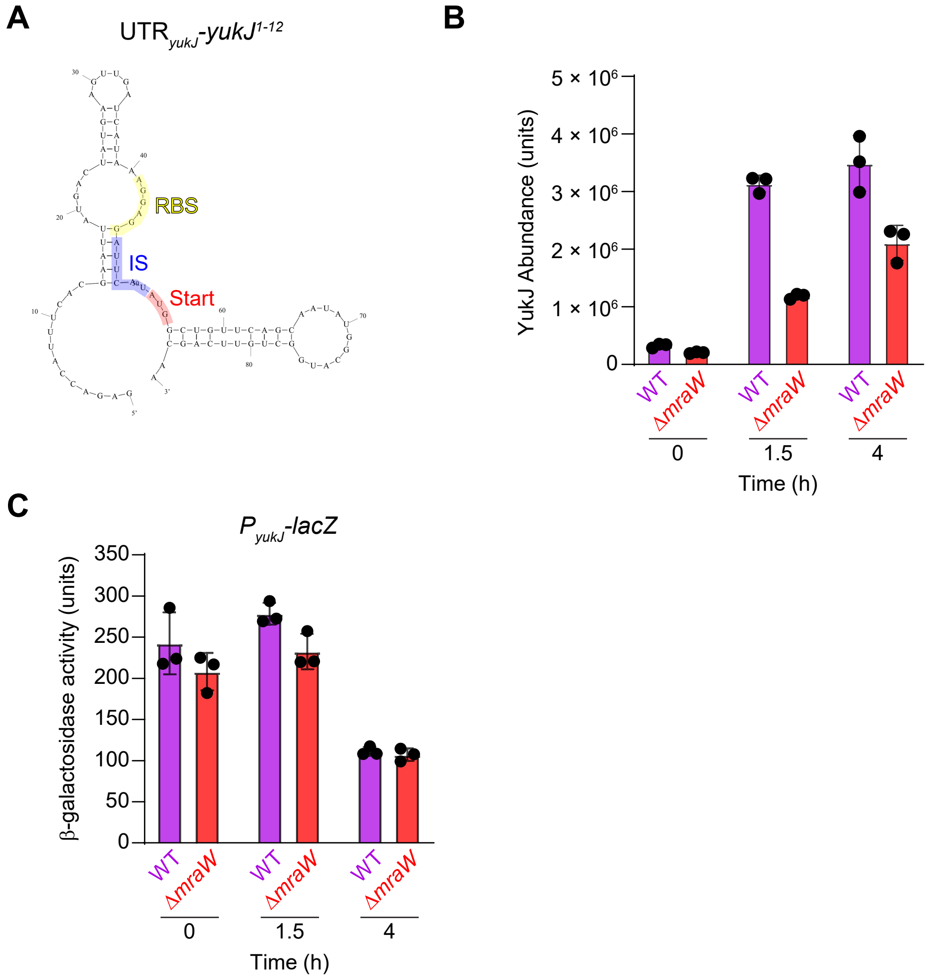

Figure S7. YukJ translation is dependent on MraW-modified ribosomes. (A) Mfold predicted structure of the 5'UTR and first 12 codons of *yukJ*. (B) YukJ abundance measured by mass spectrometry in the presence (purple) or absence (red) of *mraW* at the indicated time points. Bars represent mean (n = 3 biological replicates); errors: S.D. Strains: PY79 and ZP1. (C) β-galactosidase activity arising from a transcriptional fusion of the promoter of *yukJ* to *lacZ* in the presence (purple) or absence (red) of *mraW* at the indicated time points. Bars represent mean (n = 3 biological replicates); errors: S.D. Strains: ZP546 and ZP550.

bioRxiv preprint doi: https://doi.org/10.64898/2026.04.20.719615; this version posted April 20, 2026. The copyright holder for this preprint (which was not certified by peer review) is the author/funder. This article is a US Government work. It is not subject to copyright under 17 USC 105 and is also made available for use under a CC0 license.

Table S1. *B. subtilis s*trains used in this study.

Strain	Genotype	Reference
PY79	Prototrophic derivative of *B. subtilis* 168	[1]
KP73	Δ*spoIVA*::*kan*	[2]
IT895	Δ*spoIVA*::*kan* Δ*cmpA*::*erm*	[3]
JPC282	Δ*spoIVA*::*erm* Δ*spoVID*::*kan*	This study
ZP44	Δ*spoIVA*::*kan* Δ*mraW*::*erm*	This study
KR394	Δ*spoIVA*::*kan thrC*::*spoIVA spec*	[4]
ZP47	Δ*spoIVA*::*kan thrC*::*spoIVA*^{L59P} *spec*	This study
ZP50	Δ*spoIVA*::*kan* Δ*cmpA*::*erm thrC*::*spoIVA*^{L59P} *spec*	This study
ZP53	Δ*spoIVA*::*erm* Δ*spoVID*::*kan thrC*::*spoIVA*^{L59P} *spec*	This study
ZP56	Δ*spoIVA*::*kan* Δ*mraW*::*erm thrC*::*spoIVA*^{L59P} *spec*	This study
KR160	*thrC::gfp-spoIVA spec*	[4]
ZP35	Δ*spoIVA*::*kan amyE*::*spoIVA*^{L59P} *cat thrC*::*gfp-spoIVA*^{L59P} *spec*	This study
ZP77	Δ*spoIVA*::*kan thrC*::*spoIVA spec* Δ*mraW*::*erm*	This study
KR367	Δ*spoIVA*::*kan thrC*::*spoIVA*^{K30A} *spec*	[3]
ZP62	Δ*spoIVA*::*kan* Δ*mraW*::*erm thrC*::*spoIVA*^{K30A} *spec*	This study
JPC221	Δ*spoIVA*::*kan thrC*::*spoIVA*$^{T70A,\ T71A}$ *spec*	[5]
ZP65	Δ*spoIVA*::*kan* Δ*mraW*::*erm thrC*::*spoIVA*$^{T70A,\ T71A}$ *spec*	This study
JB103	Δ*spoIVA*::*kan thrC*::*spoIVA*^{D97A} *spec*	[6]
ZP59	Δ*spoIVA*::*kan* Δ*mraW*::*erm thrC*::*spoIVA*^{D97A} *spec*	This study
ZP134	Δ*spoIVA*::*kan thrC*::*spoIVA spec* Δ*mraW*::*erm amyE*::*mraW cat*	This study
ZP231	Δ*spoIVA*::*kan thrC*::*spoIVA spec* Δ*mraW*::*erm amyE*::*mraW*^{D52A} *cat*	This study
ZP233	Δ*spoIVA*::*kan thrC*::*spoIVA spec* Δ*mraW*::*erm amyE*::*mraW*^{D100A} *cat*	This study
ZP137	Δ*spoIVA*::*kan* Δ*mraW*::*erm thrC*::*spoIVA*^{L59P} *spec amyE*::*mraW cat*	This study
ZP239	Δ*spoIVA*::*kan thrC*::*spoIVA*^{L59P} *spec* Δ*mraW*::*erm amyE*::*mraW*^{D52A} *cat*	This study
ZP241	Δ*spoIVA*::*kan thrC*::*spoIVA*^{L59P} *spec* Δ*mraW*::*erm amyE*::*mraW*^{D100A} *cat*	This study
ZP221	Δ*spoIVA*::*kan thrC*::*spoIVA spec* Δ*yabC*::*erm*	This study
ZP287	Δ*spoIVA*::*kan* Δ*mraW*::*erm thrC*::*spoIVA spec* Δ*yabC*::*erm*::*cat*	This study
ZP215	Δ*spoIVA*::*kan thrC*::*spoIVA*^{L59P} *spec* Δ*yabC*::*erm*	This study
ZP283	Δ*spoIVA*::*kan* Δ*mraW*::*erm thrC*::*spoIVA*^{L59P} *spec* Δ*yabC*::*erm*::cat	This study
ZP249	Δ*spoIVA*::*kan thrC*::*spoIVA*$^{T70A,\ T71A}$ *spec* Δ*yabC*::*erm*	This study
ZP285	Δ*spoIVA*::*kan* Δ*mraW*::*erm thrC*::*spoIVA*$^{T70A,\ T71A}$ *spec* Δ*yabC*::*erm*::*cat*	This study
ZP1	Δ*mraW*::*erm*	This study
ZP89	Δ*spoIVA*::*kan* Δ*mraW*::*erm thrC*::*spoIVA spec amyE*::P_{spoIVA}-*mraW cat*	This study

bioRxiv preprint doi: https://doi.org/10.64898/2026.04.20.719615; this version posted April 20, 2026. The copyright holder for this preprint (which was not certified by peer review) is the author/funder. This article is a US Government work. It is not subject to copyright under 17 USC 105 and is also made available for use under a CC0 license.

ZP83	Δ*spoIVA*::*kan* Δ*mraW*::*erm thrC*::$spoIVA^{L59P}$ *spec amyE*::P_{spoIVA}-*mraW cat*	This study
ZP107	Δ*spoIVA*::*kan* Δ*mraW*::*erm thrC*::*spoIVA spec amyE*::P_{cotR}-*mraW cat*	This study
ZP104	Δ*spoIVA*::*kan* Δ*mraW*::*erm thrC*::$spoIVA^{L59P}$ *spec amyE*::P_{cotR} -*mraW cat*	This study
ZP385	Δ*spoIVA*::*kan sacA*::$spoIVA^{T70A,\ T71A}$ *cat*::*spec amyE*::P_{cmpA}-*cmpA-lacZ cat*	This study
ZP389	Δ*spoIVA*::*kan 1`*::$spoIVA^{T70A,\ T71A}$ *cat*::*spec* Δ*mraW*::*erm amyE*::P_{cmpA}-*cmpA-lacZ cat*	This study
ZP383	Δ*spoIVA*::*kan sacA*::$spoIVA^{T70A,\ T71A}$ *cat*::*spec amyE*::P_{cmpA}-*lacZ cat*	This study
ZP387	Δ*spoIVA*::*kan sacA*::$spoIVA^{T70A,\ T71A}$ *cat*::*spec* Δ*mraW*::*erm amyE*::P_{cmpA}-*lacZ cat*	This study
SE230	*amyE*::P_{cmpA}-*cmpA-lacZ cat*	[7]
ZP313	*amyE*::P_{cmpA}-*cmpA-lacZ cat* Δ*mraW*::*erm*	This study
SE222	*amyE*::P_{cmpA}-*lacZ cat*	[7]
ZP311	*amyE*::P_{cmpA}-*lacZ cat* Δ*mraW*::*erm*	This study
IT686	*sacA*::P_{sigA}-*cmpA cat*	[3]
ZP116	Δ*mraW*::*erm sacA*::P_{sigA}-*cmpA cat*	This study
ZP315	Δ*spoIVA*::*kan thrC*::*spoIVA spec sacA*::P_{sigA}-*cmpA cat*	This study
ZP321	Δ*spoIVA*::*kan thrC*::*spoIVA spec amyE*::P_{sigA}-*cmpA cat* Δ*mraW*::*erm*	This study
ZP317	Δ*spoIVA*::*kan thrC*::$spoIVA^{T70A,\ T71A}$ *spec amyE*::P_{sigA}-*cmpA cat*	This study
ZP319	Δ*spoIVA*::*kan thrC*::$spoIVA^{T70A,\ T71A}$ *spec amyE*::P_{sigA}-*cmpA cat* Δ*mraW*::*erm*	This study
ZP441	*amyE*::P_{cmpA}-$cmpA^{1\text{-}30}$-*lacZ cat*	This study
ZP446	*amyE*::P_{cmpA}-$cmpA^{1\text{-}30}$-*lacZ cat* Δ*mraW*::*erm*	This study
ZP439	*amyE*::P_{cmpA}-$cmpA^{1\text{-}20}$-*lacZ cat*	This study
ZP444	*amyE*::P_{cmpA}-$cmpA^{1\text{-}20}$-*lacZ cat* Δ*mraW*::*erm*	This study
ZP437	*amyE*::P_{cmpA}-$cmpA^{1\text{-}10}$-*lacZ cat*	This study
ZP442	*amyE*::P_{cmpA}-$cmpA^{1\text{-}10}$-*lacZ cat* Δ*mraW*::*erm*	This study
ZP463	*amyE*::P_{cmpA}-$cmpA^{1\text{-}20}$ (-1 frameshift)-*lacZ cat*	This study
ZP469	*amyE*::P_{cmpA}-$cmpA^{1\text{-}20}$ (-1 frameshift)-*lacZ cat* Δ*mraW*::*erm*	This study
ZP560	*sacA*::P_{sigA}-IS*-*cmpA cat*	This study
ZP562	*sacA*::P_{sigA}-IS*-*cmpA cat* Δ*mraW*::*erm*	This study
ZP451	*amyE*::P_{cmpA}-$cmpA^{1\text{-}4}$-*lacZ cat*	This study
ZP457	*amyE*::P_{cmpA}-$cmpA^{1\text{-}4}$-*lacZ cat* Δ*mraW*::*erm*	This study
ZP473	*amyE*::P_{cmpA}-$cmpA^{1\text{-}6}$-*lacZ cat*	This study
ZP481	*amyE*::P_{cmpA}-$cmpA^{1\text{-}6}$-*lacZ cat* Δ*mraW*::*erm*	This study
ZP503	*amyE*::P_{cmpA}-$cmpA^{stem\text{-}swap}$-*lacZ cat*	This study
ZP497	*amyE*::P_{cmpA}-$cmpA^{stem\text{-}swap}$-*lacZ cat* Δ*mraW*::*erm*	This study
ZP532	*amyE*::P_{cmpA}-$cmpA^{1\text{-}6}$ (codons 4*/5*)-*lacZ cat*	This study
ZP534	*amyE*::P_{cmpA}-$cmpA^{1\text{-}6}$ (codons 4*/5*)-*lacZ cat* Δ*mraW*::*erm*	This study

bioRxiv preprint doi: https://doi.org/10.64898/2026.04.20.719615; this version posted April 20, 2026. The copyright holder for this preprint (which was not certified by peer review) is the author/funder. This article is a US Government work. It is not subject to copyright under 17 USC 105 and is also made available for use under a CC0 license.

ZP568	*amyE*::P_{cmpA}-*cmpA*$^{1\text{-}6}$ (stem strong)-*lacZ cat*	This study
ZP570	*amyE*::P_{cmpA}-*cmpA*$^{1\text{-}6}$ (stem strong)-*lacZ cat* Δ*mraW*::*erm*	This study
ZP507	*amyE*::P_{isp}-*lacZ cat*	This study
ZP513	*amyE*::P_{isp}-*lacZ cat* Δ*mraW*::*erm*	This study
ZP509	*amyE*::P_{isp} (stem-swap)-*lacZ cat*	This study
ZP515	*amyE*::P_{isp} (stem-swap)-*lacZ cat* Δ*mraW*::*erm*	This study
ZP577	*amyE*::P_{isp} (IS*)-*lacZ cat*	This study
ZP583	*amyE*: P_{isp} (IS*)-*lacZ cat* Δ*mraW*::*erm*	This study
ZP572	*amyE*::P_{spoIIQ}-*T7 pol spec sacA*::P_{T7}-NEB5'UTR-*gfp cat*	This study
ZP574	*amyE*::P_{spoIIQ}-*T7 pol spec sacA*::P_{T7}-NEB5'UTR-*gfp cat* Δ*mraW*::*erm*	This study
ZP405	Δ*spoIVA*::*kan sacA*::*spoIVA*$^{T70A,\ T71A}$ *cat*::*spec amyE*::P_{spoVID}-*spoVID-lacZ cat*	This study
ZP410	Δ*spoIVA*::*kan sacA*::*spoIVA*$^{T70A,\ T71A}$ *cat*::*spec amyE*::P_{spoVID}-*spoVID-lacZ cat* Δ*mraW*::*erm*	This study
ZP339	*amyE*::P_{spoVID}-*spoVID-lacZ cat*	This study
ZP345	*amyE*::P_{spoVID}-*spoVID-lacZ cat* Δ*mraW*::*erm*	This study
ZP363	*amyE*::P_{sigA}-*lacZ cat*	This study
ZP361	*amyE*::P_{sigA}-*lacZ cat* Δ*mraW*::*erm*	This study
ZP523	*amyE*::P_{sigA}-*cmpA-gfp cat*	This study
ZP525	*amyE*::P_{sigA}-*cmpA-gfp cat* Δ*mraW*::*erm*	This study
ZP429	Δ*spoIVA*::*kan sacA*::*spoIVA cat*::*spec*	This study
ZP435	Δ*spoIVA*::*kan sacA*::*spoIVA cat*::*spec* Δ*mraW*::*erm*	This study
ZP379	Δ*spoIVA*::*kan sacA*::*spoIVA*$^{T70A,\ T71A}$ *cat*::*spec*	This study
ZP381	Δ*spoIVA*::*kan sacA*::*spoIVA*$^{T70A,\ T71A}$ *cat*::*spec* Δ*mraW*::*erm*	This study
ZP546	*amyE*::P_{yukJ}-*lacZ cat*	This study
ZP550	*amyE*::P_{yukJ}-*lacZ cat* Δ*mraW*::*erm*	This study
ZP511	*amyE*::P_{isp} (stem strong)-*lacZ cat*	This study
ZP517	*amyE*: P_{isp} (stem strong)-*lacZ cat* Δ*mraW*::*erm*	This study

REFERENCES

1. Youngman, P., Perkins, J.B., and Losick, R. (1984). Construction of a cloning site near one end of Tn917 into which foreign DNA may be inserted without affecting transposition in *Bacillus subtilis* or expression of the transposon-borne erm gene. Plasmid *12*, 1-9. 10.1016/0147-619x(84)90061-1.
2. Price, K.D., and Losick, R. (1999). A four-dimensional view of assembly of a morphogenetic protein during sporulation in *Bacillus subtilis*. J Bacteriol *181*, 781-790. 10.1128/JB.181.3.781-790.1999.
3. Tan, I.S., Weiss, C.A., Popham, D.L., and Ramamurthi, K.S. (2015). A Quality-Control Mechanism Removes Unfit Cells from a Population of Sporulating Bacteria. Dev Cell *34*, 682-693. 10.1016/j.devcel.2015.08.009.

bioRxiv preprint doi: https://doi.org/10.64898/2026.04.20.719615; this version posted April 20, 2026. The copyright holder for this preprint (which was not certified by peer review) is the author/funder. This article is a US Government work. It is not subject to copyright under 17 USC 105 and is also made available for use under a CC0 license.

4. Ramamurthi, K.S., and Losick, R. (2008). ATP-driven self-assembly of a morphogenetic protein in *Bacillus subtilis*. Mol Cell *31*, 406-414. 10.1016/j.molcel.2008.05.030.
5. Castaing, J.P., Nagy, A., Anantharaman, V., Aravind, L., and Ramamurthi, K.S. (2013). ATP hydrolysis by a domain related to translation factor GTPases drives polymerization of a static bacterial morphogenetic protein. Proc Natl Acad Sci U S A *110*, E151-160. 10.1073/pnas.1210554110.
6. Delerue, T., Anantharaman, V., Gilmore, M.C., Popham, D.L., Cava, F., Aravind, L., and Ramamurthi, K.S. (2022). Bacterial developmental checkpoint that directly monitors cell surface morphogenesis. Dev Cell *57*, 344-360 e346. 10.1016/j.devcel.2021.12.021.
7. Ebmeier, S.E., Tan, I.S., Clapham, K.R., and Ramamurthi, K.S. (2012). Small proteins link coat and cortex assembly during sporulation in *Bacillus subtilis*. Mol Microbiol *84*, 682-696. 10.1111/j.1365-2958.2012.08052.x.
